# Cell wall components of gut commensal bacteria stimulate peritrophic matrix formation in malaria vector mosquitoes through activation of the IMD pathway

**DOI:** 10.1371/journal.pbio.3002967

**Published:** 2025-01-06

**Authors:** Xiumei Song, Han Zhou, Jingwen Wang

**Affiliations:** 1 State Key Laboratory of Genetic Engineering, School of Life Sciences, Department of Infectious Diseases, Zhongshan Hospital, Fudan University, Shanghai, China; 2 Ministry of Education Key Laboratory for Biodiversity Science and Ecological Engineering, School of Life Sciences, Fudan University, Shanghai, China; Institut Pasteur, FRANCE

## Abstract

The peritrophic matrix (PM) acts as a physical barrier that influences the vector competence of mosquitoes. We have previously shown that gut microbiota promotes PM formation in *Anopheles stephensi*, although the underlying mechanisms remain unclear. In this study, we identify that the cell wall components of gut commensal bacteria contribute to PM formation. Oral administration of primary cell wall components from both gram-positive and gram-negative bacteria, such as diaminopimelic acid-peptidoglycan (DAP-PGN), lysine-peptidoglycan (Lys-PGN), and lipopolysaccharides (LPS), to mosquitoes, after depleting their gut microbiota with antibiotics, restores the down-regulated expression of the *peritrophin1* (*Per1*) gene, which encodes a structural protein of the PM. Moreover, this administration rescues PM formation upon blood ingestion. PGN and LPS are well-known ligands of innate immune signaling pathways in animals. In mosquitoes, the Toll and IMD (immune deficiency) pathways are the 2 major innate immune signaling pathways. We next knocked down the expression of 2 receptors, *Pgrp-s1* and *Pgrp-lc*, as well as 2 transcription factors, *Rel1* and *Rel2*, which are involved in the Toll and IMD pathways, respectively. Double knockdown of *Pgrp-s1* and *Pgrp-lc*, or *Rel1* and *Rel2*, compromised *Per1* expression. Additionally, through dual-luciferase assays and supershift electrophoretic mobility shift assays (EMSAs), we identified a 15-bp binding motif (ATAGACACGAGCACC) for Rel1 and Rel2 in the *Per1* promoter region. To further explore the role of individual Toll and IMD pathways in the regulation of *Per1* expression, we specifically inhibited the activity of each pathway. While inhibition of the Toll pathway by knocking down *Pgrp-s1* or *Rel1* did not affect *Per1* expression, knockdown of *Pgrp-lc* or *Rel2* in the IMD pathway significantly down-regulated *Per1* expression. These findings suggest that the IMD pathway plays a major role in regulating *Per1* expression in *An*. *stephensi*. In summary, our study uncovers a novel role for bacterial cell wall components in regulating PM formation through activation of mosquito immune signaling pathways.

## Abstract 2

How does the mosquito gut microbiota influence the capacity to transmit pathogens? This study shows that bacterial cell wall components specifically activate the IMD pathway, and downstream NF-κB transcription factors drive the formation of peritrophic matrix, a physical barrier that influences the vector competence.

## Introduction

Malaria, which is transmitted through the bites of *Anopheles* mosquitoes, poses a significant threat to human health. During the infection in mosquitoes, *Plasmodium* parasites first undergo sexual reproduction in the mosquito midgut, where they develop into ookinetes. These ookinetes then traverse the peritrophic matrix (PM) and epithelial cells and transform into oocysts upon reaching the basal lamina [1]. Throughout this process, PM serves as the first physical barrier encountered by the invading parasites [2–4]. The PM is an acellular layer that forms when mosquitoes ingest a blood meal. It lines the ingested blood bolus and protect the midgut epithelium by separating the blood bolus from the gut epithelial cells [5–7]. The PM is composed of chitin, proteins, and glycoproteins. Chitin serves as the structural scaffold for the PM, providing a platform for proteins to bind [8,9]. Peritrophins, which are mucin linker proteins, play a crucial role in PM formation. They contain a chitin-binding domain (CBD) that enables them to interact with chitin [10,11]. Mucins are glycosylated proteins that lack a CBD domain but still contribute to PM formation by interacting with peritrophins [12].

The PM play distinct roles in influencing pathogen infection in disease-transmitting vectors. In *Anopheles* mosquitoes, the PM helps defend against *Plasmodium* infection by blocking the parasite from crossing the midgut epithelium [2]. Elimination or impairment of PM structure increases the susceptibility of *Anopheles* to *Plasmodium* infection [13,14]. In *Ixodes scapularis*, infection with *Anaplasma phagocytophilum*, the causative agent of human granulocytic anaplasmosis, disrupts the homeostasis of the gut microbiota, leading to impairment of PM structure and facilitating infection [15]. In tsetse flies, newly enclosed adults are more susceptible to trypanosome parasites than mature adults (1 week old) due to the absence of PM [16]. In the sand fly *Phlebotomus papatasi*, knockdown of *PpPer1*, a protein involved in PM formation, results in increased *Leishmania* load [17]. Conversely, it has also been reported that PM protects *Leishmania* by creating a barrier that prevents proteolytic damage to the parasite in *Phlebotomus papatasi* [18]. Similarly, the PM in *Ixodes scapularis* facilitates the colonization of *Borrelia burgdorferi*, the agent of Lyme disease [19]. Impairment of PM in *Aedes aegypti* reduces the infectivity of *Plasmodium gallinaceum*, as well as Zika and dengue virus [20,21]. Although the role of the PM in pathogen infection varies by pathogen, it is pivotal in determining the capacity of vectors to transmit diseases.

The factors influencing PM formation and integrity in most hematophagous vectors remain unclear. Symbiotic bacteria are known to play crucial roles in both stimulating PM formation and maintaining its integrity. In *Anopheles* mosquitoes and tsetse flies, elimination of symbiotic bacteria through antibiotic treatment compromises PM formation [13,22,23]. In *Anopheles* mosquito, the gut commensal bacterium *Pseudomonas alcaligenes* helps maintain PM integrity by degrading the PM-toxic tryptophan metabolite 3-hydroxykynurenine [14]. In ticks, the dysbiosis of the gut microbiota down-regulates STAT expression, leading to reduced expression of PM-related genes [19]. Similarly, in *Drosophila*, oral administration of gram-negative bacterium *Erwinia carotovora 15* induces the expression of *drosocrystallin*, a chitin-binding protein that contributes to PM formation [24]. Despite these findings, the mechanisms by which bacteria regulate PM formation remain poorly understood.

In this study, we screened the cell components of midgut commensal bacteria in *An*. *stephensi* and found that the bacterial cell wall components, PGN and LPS, stimulate the expression of *Per1*, leading to PM formation. Mechanistic analysis showed that the presence of PGNs/LPS in the midgut activates the IMD and Toll immune signaling pathways. This activation causes NF-κB transcription factors, Rel1 and Rel2, to bind to the *Per1* promoter region (−289 bp to −275 bp). Mutation of these 15 nucleotides abolishes the binding capacity of Rel1 and Rel2, thereby blocking *Per1* transcription. Furthermore, we demonstrate that the IMD pathway plays a predominant role in stimulating *Per1* expression. In summary, our results reveal an uncharacterized role of bacterial cell wall components in stimulating PM formation through immune signaling pathways.

## Results

### Bacterial cells stimulate PM formation

**Fig 1:**
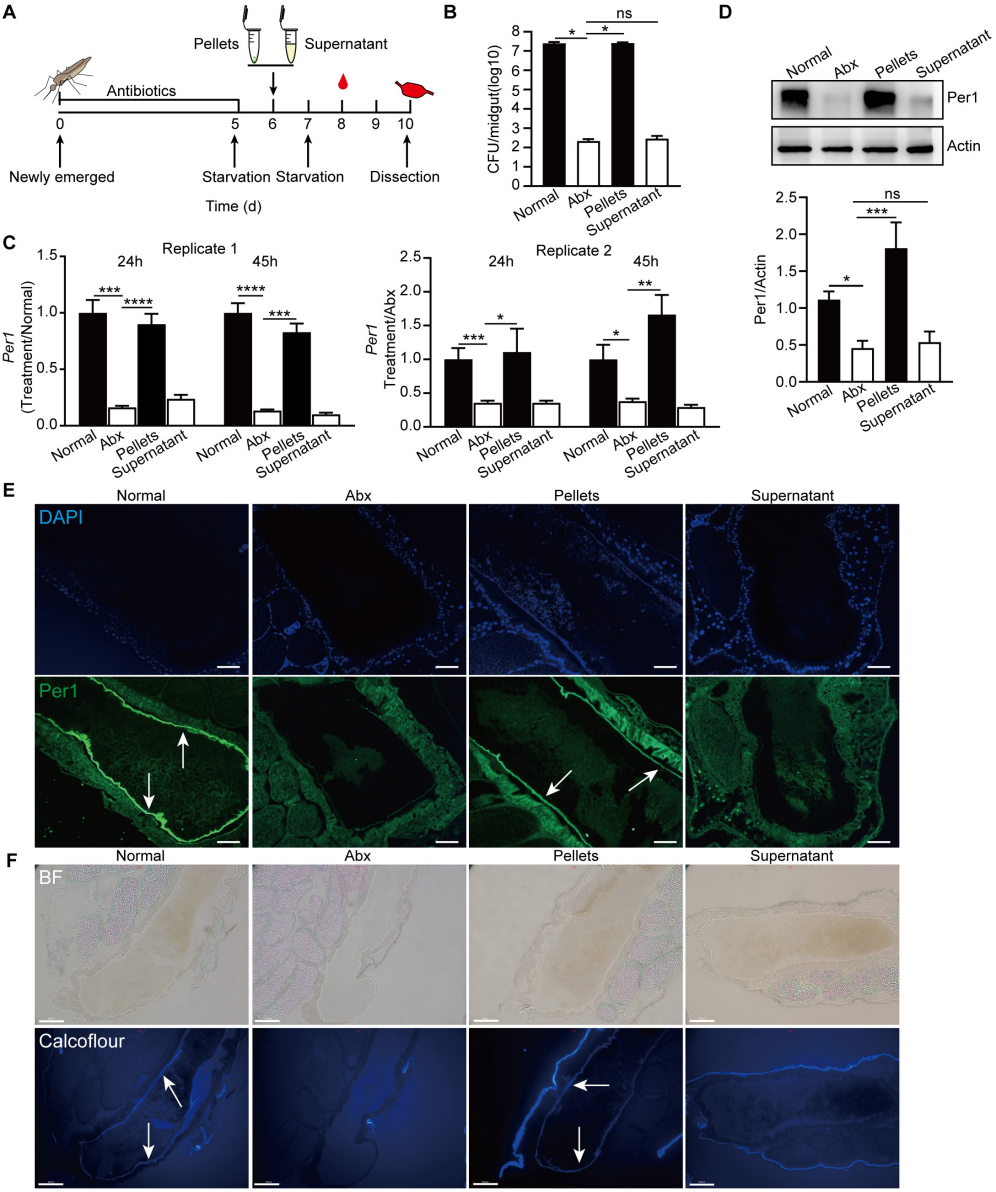
Identification of the effective component of *E*. *hormaechei* in promoting PM formation. (A) Schematic overview of the study design. (B) Bacterial loads in the midgut of normal mosquitoes and mosquitoes with different treatments at 45 h post a blood meal. Abx, antibiotic-treated mosquitoes; Pellets, Abx mosquitoes supplemented with cell pellets of *E*. *hormaechei*; Supernatant, Abx mosquitoes supplemented with cell supernatant of *E*. *hormaechei*. (C, D) The levels of *Per1* mRNA (C) and protein (D) in the midgut of the same treated mosquitoes as in (B). The expression level of *Per1* was normalized to *S7*. The relative expression level of *Per1* in treated mosquitoes was normalized to gene expression in Normal mosquitoes. Quantification of Per1 intensity was shown on the panel below in (D). Data are presented as mean ± SEM (*n* = 5 in B, *n* = 8~10 in C, and *n* = 5 in D). (E) Immunofluorescent staining of Per1 (green) in the midgut of the same treated mosquitoes as in (B) at ×200 magnification. Nuclei were stained with DAPI (blue). (F) Calcofluor white staining PM structure in the midgut of the same treated mosquitoes as in (B) at ×200 magnification. Scale bars, 50 μm. Significance was determined by one-way ANOVA followed by Dunnett’s multiple comparison test. *, *P* < 0.05, **, P < 0.01, ***, *P* < 0.001, ****, *P* < 0.0001. The data underlying this figure can be found in S1 Data. The uncropped blots are included in S1 Raw Images. PM, peritrophic matrix.

In our previous studies, we demonstrated that *Enterobacter hormaechei*, isolated from our colony mosquitoes, has the ability to stimulate PM formation in *An*. *stephensi* [22]. To investigate which component of commensal bacterium stimulates PM formation, we administrated mosquitoes, in which gut commensal bacteria were reduced by antibiotics (Abx) treatment, with the supernatant and cell pellets of the overnight culture of *E*. *hormaechei* (Fig 1A). The efficacy of bacterial clearance by antibiotics was evaluated by qPCR and plate culture methods (S1 Fig). In mosquitoes fed with *E*. *hormaechei* pellets, the bacterial load reached a similar level as that in normal mosquitoes, around 10^7^–10^8^ colony-forming units (CFU), at 45 h after blood feeding. However, the bacterial load in mosquitoes treated with the bacterial supernatant and Abx-treated mosquitoes remained at the same level (Fig 1B). We next analyzed the mRNA and protein levels of *Per1*, a gene involved in PM formation, in these mosquitoes. Administration of *E*. *hormaechei* pellets significantly up-regulated the expression of *Per1* mRNA at 24 and 45 h after blood feeding, and Per1 protein at 45 h after blood feeding, reaching levels comparable to those found in the normal mosquito midgut. However, treatment with bacterial supernatant failed to stimulate *Per1* expression (Fig 1C and 1D). Consistent with these findings, fully formed PMs were observed by immunofluorescent staining against Per1 and calcofluor staining in mosquitoes treated with bacterial pellets and in normal mosquitoes at 45 h after blood meal, while PM formation was impaired in mosquitoes treated with bacterial supernatant or Abx-treated mosquitoes (Fig 1E and 1F). Based on these results, we conclude that bacterial secretion does not play a role in promoting PM formation. Instead, components present in the bacterial cells are responsible for stimulating PM formation in mosquitoes.

### The bacterial cell wall promotes PM formation

**Fig 2:**
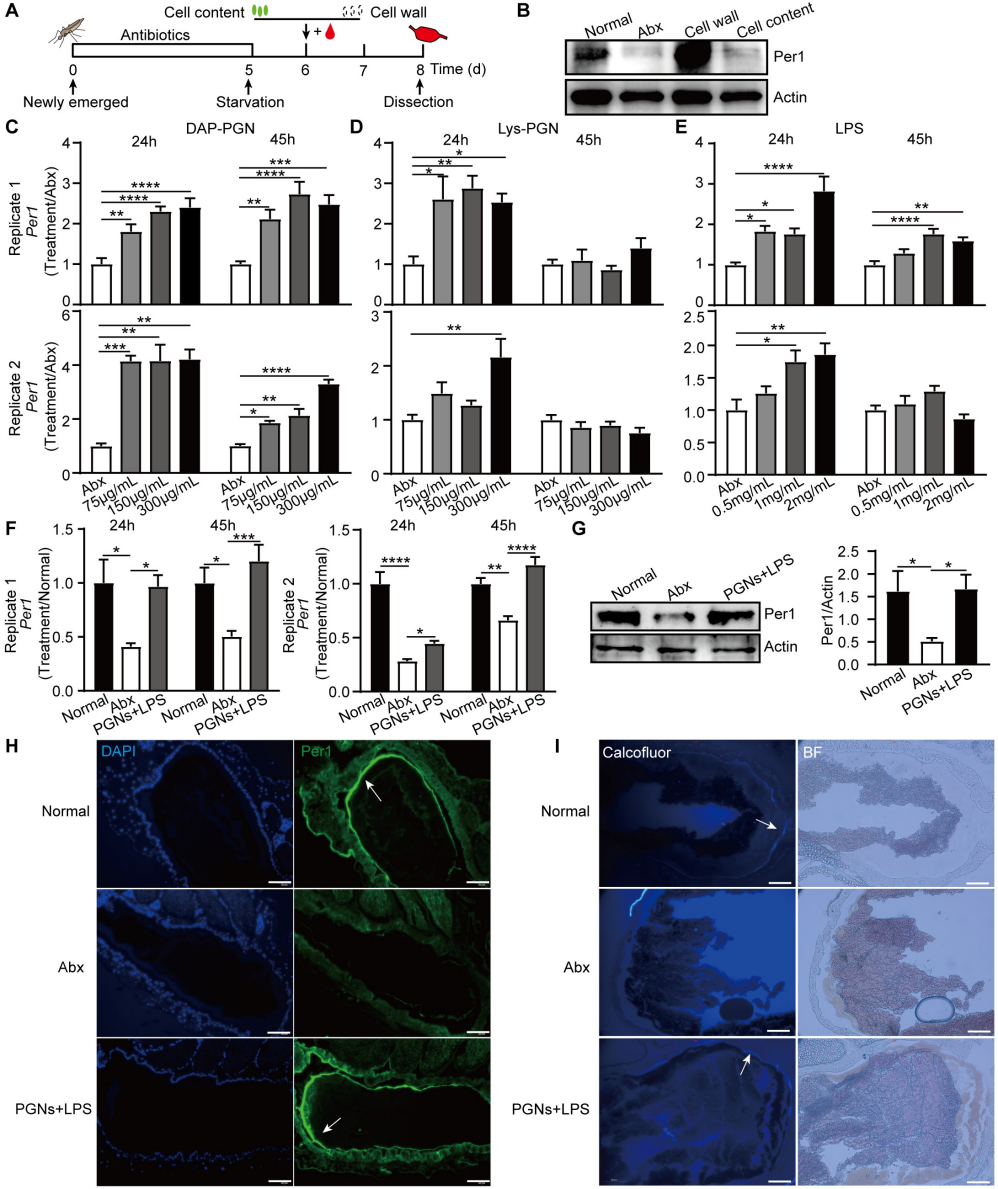
Identification of the bacterial cell component in PM formation. (A) Schematic overview of the study design. (B) Western blot of Per1 in the midguts of normal, Abx mosquitoes and Abx mosquitoes treated with cell wall and cell content at 45 h post a blood meal. (C–E) The expression levels of *Per1* of mosquitoes orally supplemented with serial concentrations of DAP-PGN (C), Lys-PGN (D), LPS (E), at 24 and 45 h post a blood meal. (F, G) The expression levels of *Per1* mRNA (F) and protein (G) in the midgut of Normal, Abx, and Abx mosquitoes treated with the mixture of DAP-PGN, Lys-PGN, and LPS (PGNs+LPS) at 24 and 45 h post a blood meal. The expression level of the target gene was normalized to *S7*. The relative expression level of *Per1* was normalized to gene expression in Abx or Normal mosquitoes. Quantification of Per1 intensity was shown on the right panel in (G). Data are presented as mean ± SEM (*n* = 8~10 in C, *n* = 7~10 in D, *n* = 8~10 in E, and *n* = 10 in F and *n* = 4 in G). (H) Immunofluorescent staining of Per1 (green) in midguts of Normal, Abx, and PGNs+LPS treated mosquitoes at 45 h post a blood meal at ×200 magnification. Nuclei were stained with DAPI (blue). (I) Calcofluor white staining PM structure in the midgut of Normal, Abx, and PGNs+LPS treated mosquitoes at 45 h post a blood meal at ×200 magnification. Scale bars, 50 μm. Significance was determined by one-way ANOVA followed by Dunnett’s multiple comparison test. *, *P* < 0.05, **, *P* < 0.01, ***, *P* < 0.001, ****, *P* < 0.0001. The data underlying this figure can be found in S1 Data. The uncropped blots are included in S1 Raw Images. DAP-PGN, diaminopimelic acid-peptidoglycan; LPS, lipopolysaccharide; Lys-PGN, lysine-peptidoglycan; PM, peritrophic matrix.

A bacterial cell typically consists of a cell wall that surrounds an internal matrix called the cytoplasm [25]. To identify the bacterial effectors responsible for promoting PM formation, we isolated the bacterial cell wall and intracellular contents from an overnight culture of *E*. *hormaechei*. These isolated components were individually administered to Abx-treated mosquitoes through a blood meal (Fig 2A). Supplementation of the bacterial cell wall increased the protein level of Per1, indicating its role in stimulating PM formation. However, the intracellular contents had no influence on Per1 protein level (Fig 2B).

Peptidoglycan, including DAP-PGN and Lys-PGN, as well as LPS, are major components of the bacterial cell wall [26]. We next fed Abx-treated mosquitoes with blood containing varying concentrations of DAP-PGN, Lys-PGN, or LPS individually, and analyzed *Per1* expression levels of at 24 h and 45 h post-blood feeding. All 3 components stimulated *Per1* expression at 24 h post-blood feeding, while DAP-PGN continued to elevate *Per1* expression at 45 h (Fig 2C–2E), suggesting that DAP-PGN elicits a more sustained induction of *Per1* than other cell wall components. Since the induction of *Per1* by LPS may be due to the PGN contamination [27,28], we next treated LPS with mutanolysin, a PGN lytic enzyme [27], and then fed mosquitoes with the treated LPS via blood. Again, significant up-regulation of *Per1* expression was observed in both mutanolysin-untreated and treated LPS mosquitoes (S2 Fig), suggesting that LPS is involved in the stimulation of PM formation. Considering that the mosquito gut microbiota consists of both gram-negative and gram-positive bacteria [29], we combined all 3 cell wall components and fed the mixture to Abx-treated mosquitoes via blood. As expected, oral administration of the PGNs/LPS mixture restored the mRNA and protein levels of Per1 to similar levels as those observed in normal mosquitoes (Fig 2F and 2G). Additionally, intact PM formation was observed in the midgut of mosquitoes orally supplemented with the mixture (Fig 2H and 2I). Taken together, these results indicate that the components of the bacterial cell wall, including DAP-PGN, Lys-PGN, and LPS, play a crucial role in stimulating PM formation.

### The PGNs/LPS mixture stimulates *Per1* expression prior to blood feeding

**Fig 3:**
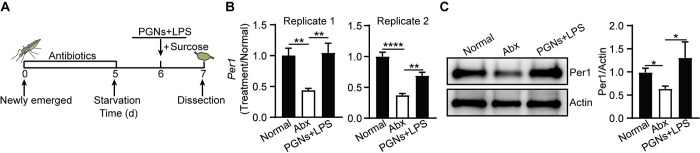
The influence of PGNs and LPS on *Per1* expression before blood feeding. (A) Schematic overview of the study design. (B, C) The levels of *Per1* mRNA (B) and protein (C) in the midgut of Normal, Abx, Abx mosquitoes treated with DAP-PGN/Lys-PGNs/LPS mixture via sugar meal for 24 h. The expression level of *Per1* was normalized to *S7*. The relative expression levels of *Per1* in Abx, Abx supplemented with PGNs+LPS mosquitoes were normalized to gene expression in Normal mosquitoes. Quantification of Per1 intensity was shown on the right panel in (C). Data are presented as mean ± SEM (*n* = 7~10 in B, *n* = 4 in C). Significance was determined by one-way ANOVA followed by Dunnett’s multiple comparison test. *, *P* < 0.05, **, *P* < 0.01, ****, *P* < 0.0001. The data underlying this figure can be found in S1 Data. The uncropped blots are included in S1 Raw Images. DAP-PGN, diaminopimelic acid-peptidoglycan; LPS, lipopolysaccharide; Lys-PGN, lysine-peptidoglycan.

Peritrophins are stored in the secretory vesicles of midgut epithelial cells before blood feeding and are released into the gut lumen immediately after mosquitoes ingest a blood meal [30–32]. To investigate whether the elimination of gut commensal bacteria influences Per1 level before a blood meal, we analyzed the expression level of Per1 in mosquitoes that were only fed sugar (Fig 3A). As expected, the mRNA and protein levels of Per1 were reduced significantly in Abx-treated mosquitoes compared to normal mosquitoes (Fig 3B and 3C). However, when we administered the PGNs/LPS mixture to Abx-treated mosquitoes via a sugar meal, we found that the mixture induced Per1 expression at both the mRNA and protein levels even without a blood meal (Fig 3B and 3C). Additionally, 2 other PM-related genes were also regulated by bacterial cell wall components (S3 Fig) [33–35]. These results suggest that the PGNs/LPS mixture stimulates PM genes expression independently of the type of food that the mosquito ingests. Even in the absence of a blood meal, the administration of the PGNs/LPS mixture can trigger PM gene expression, indicating that the bacterial cell wall components play a direct role in stimulating PM formation in the mosquito gut.

### Per1 expression is regulated by IMD and Toll pathways in mosquito

**Fig 4:**
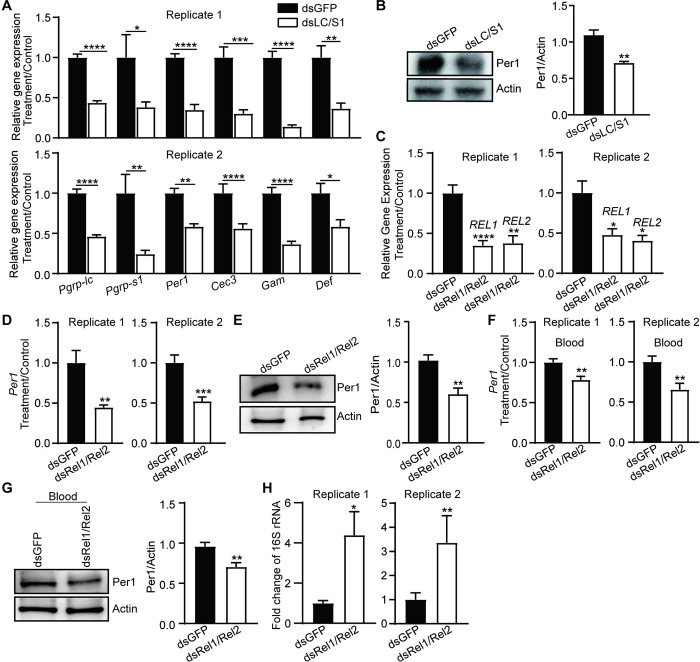
The influence of Toll and IMD pathways on *Per1* expression. (A) Relative expression level of *Per1* and immune related genes in mosquitoes treated with dsLC/S1 and dsGFP (*n* = 10). (B) Western blot of Per1 in the midgut of dsGFP and dsLC/S1-treated mosquitoes. Quantification of Per1 intensity was shown on the right panel in (B). (C) The knocking down efficiency of *Rel1* and *Rel2* of mosquitoes (dsRel1/Rel2). (D, E) The levels of *Per1* transcript (D) and protein (E) in dsRel1/Rel2 and dsGFP-treated mosquitoes prior to blood feeding. Quantification of Per1 intensity was shown on the right panel in (E). (F, G) The levels of *Per1* transcript (F) and protein (G) in dsRel1/Rel2 and dsGFP-treated mosquitoes at 45 h post blood feeding. The expression levels of the target genes were normalized to *S7*. The relative expression level of immune genes in dsRel1/Rel2-treated mosquitoes was normalized to gene expression in dsGFP-treated mosquitoes. Quantification of Per1 intensity was shown on the right panel in (G). (H) The total gut microbiota load was measured in dsRel1/Rel2 and dsGFP treated mosquitoes prior to blood feeding. All Data are presented as mean ± SEM (*n* = 9~10 in A, *n* = 3 in B, *n* = 8~9 in C, *n* = 8~9 in D, *n* = 4, *n* = 9~10 in F, *n* = 5 in G, *n* = 8~10 in H). Significance was determined by Student’s *t* test. The microbiota load was determined by Mann–Whitney test. *, *P* < 0.05, **, *P* < 0.01, ***, *P* < 0.001, ****, *P* < 0.0001. The data underlying this figure can be found in S1 Data. The uncropped blots are included in S1 Raw Images.

The cell wall components, particularly PGNs and LPS, are well-known activators of the mosquito innate immune system [36–38]. We hypothesized that the bacterial cell wall might regulate *Per1* expression through immune signaling pathways. In *Drosophila*, the peptidoglycan recognition proteins PGRP-LC and -SA are responsible for the recognition of PGNs and act as receptors for the IMD and Toll pathways, respectively [39–42]. In mosquitoes, PGRP-LC is the key receptor of IMD pathway [43], while PGRP-S1 is a putative receptor of Toll pathway [44–46]. To investigate whether the IMD and Toll pathways regulate *Per1* expression through sensing PGN and LPS, we conducted a co-silencing experiment targeting *Pgrp-lc* and -*s1* (dsLC/S1) and examined the expression level of *Per1* in mosquitoes before blood feeding. The mRNA and protein levels of Per1 were significantly reduced in mosquitoes with *Pgrp-lc* and *Pgrp-s1* double knockdown compared to those with dsGFP controls (Fig 4A and 4B). As expected, the knockdown of *Pgrp-lc* and *Pgrp-s1* inhibited the activities of the IMD and Toll signaling pathways, as indicated by the significantly reduced expression levels of antimicrobial peptides (AMPs), including *Cecropin3*, *Gambicin*, and *Defensin* (Fig 4A). The simultaneous decrease in the expression of *Per1* and AMPs in dsLC/S1 mosquitoes suggests that these genes may be regulated by common transcription factors or signaling components downstream of the IMD and Toll pathways.

The NF-κB transcription factors Rel1 and Rel2, which belong to the Toll and IMD pathways, respectively, are the primary regulators of downstream immune effector expression. To investigate whether Rel1 and Rel2 regulate *Per1* expression, we conducted a double knockdown experiment targeting *Rel1* and *Rel2* and analyzed *Per1* expression. When *Rel1* and *Rel2* were simultaneously knocked down, both the mRNA and protein levels of Per1 were significantly reduced compared to the dsGFP control, regardless of whether mosquitoes were fed sugar or blood (Fig 4C–4G). Moreover, the expression levels of *Per14* and *Fibrinogen*, which were induced by treatment with PGNs/LPS mixture in Abx mosquitoes, were also reduced in dsRel1/Rel2 mosquitoes (S4 Fig). Given that the NF-κB signaling pathways are responsible for controlling the abundance of gut microbiota that stimulates PM formation, we investigated whether the down-regulation of *Per1* expression was due to alterations in gut bacteria abundance. The bacterial load in the midgut of dsRel1/Rel2 mosquitoes was significantly increased prior to and post blood meal due to the inhibition of mosquito Toll and IMD signaling activities (Figs 4H and S4C). However, despite the increased bacterial load, the expression level of Per1 in these mosquitoes remained down-regulated, further confirming that the inhibiting Rel1 and Rel2 activity prevents the initiation of *Per1* expression. These results suggest that Rel1 and Rel2 directly regulate *Per1* expression and underscore the importance of these NF-κB transcription factors in coordinating the immune response and PM formation in mosquitoes.

### Rel1/Rel2 control the expression of *Per1* by binding to a 15 bp regulatory motif

**Fig 5:**
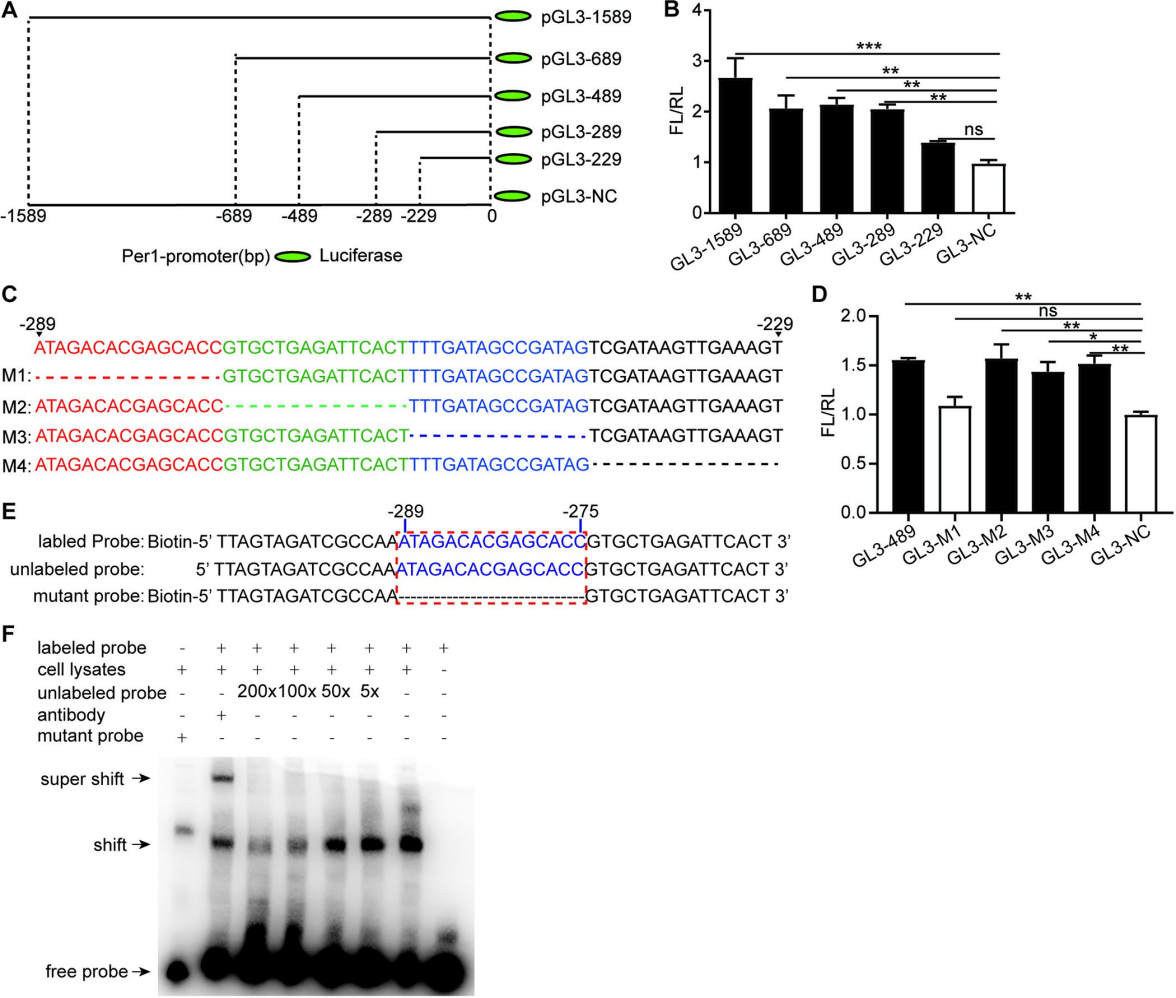
Identification of the Rel1/Rel2 binding motif in *Per1* promoter. (A) A diagram of *Per1* promoter and the truncated constructs used in the transient transfection assays. The truncations of the *Per1* promoter, which were sequentially truncated from the 5′-end of promoter region, derived from the −1,589 bp to −229 bp, were inserted into pGL3-Basic vector. The inserted promoters were followed by a firefly luciferase gene (green), thereby enabling the determination of the regulatory activity of the inserted promoters using dual luciferase reporter assay. The pGL3-Basic vector was function as control named as pGL3-NC. (B) Assessment of promoter activity for *Per1* promoter truncations together with Rel1-RHD and Rel2-RHD plasmids. (C) Schematic representation of M1, M2, M3, M4 mutants, derived from the −289 bp to −229 bp in the *Per1* promoter. M, mutation. (D) Assessment of promoter activity for *Per1* promoter mutations together with Rel1-RHD and Rel2-RHD plasmids. The activity of *Per1* promoter with different mutations was normalized to the activity of the control luciferase construct. Each experiment was performed at least 2 times independently and in duplicate. Data are presented as mean ± SEM. Significance was determined by one-way ANOVA followed by Dunnett’s multiple comparison test. *, *P* < 0.05, **, *P* < 0.01, ***, *P* < 0.001. (E) Design of probes for the EMSA assay. The boxed regions represent the regulatory sites for Rel1 and Rel2 factors. (F) Determination of the binding sites of Rel1 and Rel2 in *Per1* promoter by EMSA. The arrows indicate specific DNA-protein complex. The data underlying this figure can be found in S1 Data. The uncropped blots are included in S1 Raw Images. EMSA, electrophoretic mobility shift assay; RHD, Rel-homology domain.

Mosquito Rel1 and Rel2 contain the Rel-homology domain (RHD), which initiates the transcription of target genes. We next co-expressed the RHD domains of Rel1 and Rel2, respectively, and investigated their activity on *Per1* transcription using a dual-luciferase reporter assay. The pCMV6-Rel1-RHD and pCMV6-Rel2-RHD plasmids were co-transfected into 293T cells. The pGL3-Basic Reporter plasmids containing promoter fragment 1,589 bp upstream of the *Per1* coding sequence was transfected into 293T cells (Fig 5A). The plasmid that constitutively expresses renilla luciferase (pRLTK-renilla) was co-transfected as an internal control. At 48 h post-transfection, Rel1 and Rel2 drove the expression of the firefly luciferase reporter successfully in the presence of the −1,589 bp promoter region (Fig 5B). We next analyzed the Rel1 and Rel2 recognition region by transfecting cells with different lengths of promoter fragments ranging from −1,589 to −229 bp upstream of the coding sequence. We found that the deletion of −289 to −229 bp resulted in the loss of capability to activate the firefly luciferase reporter expression, compared to transfection with the pGL3-Basic Reporter vector (Fig 5B). This result indicates that the binding motif of Rel1 and Rel2 may exist within the −289 bp to −229 bp region.

To identify binding motifs within the minimal 61 bp region, we constructed 4 deletion mutants from −289 bp to −229 bp of the *Per1* promoter sequence and designated them as M1 (−289 bp to −275 bp deletion), M2 (−274 bp to −260 bp deletion), M3 (−259 bp to −245 bp deletion), and M4 (−244 bp to −229 bp deletion), respectively (Fig 5C). Only M1 failed to activate firefly luciferase reporter expression, compared to wild-type controls (pGL3-489), indicating that the binding motif is located at the region from −289 bp to −275 bp of *Per1* promoter (Fig 5C and 5D). To further validate this, we performed an electrophoretic mobility shift assay (EMSA). The pCMV6-Rel1-RHD and pCMV6-Rel2-RHD plasmids containing His-tag were co-transfected into 293T cells. When biotin-labeled probes were incubated with cell lysates, a shifted band was observed, indicating that Rel1 and Rel2 proteins bind to biotin-labeled probes. The band intensities gradually decreased with the addition of 5-fold, 50-fold, 100-fold, and 200-fold excess of unlabeled competitive oligonucleotide probes, suggesting that Rel1 and Rel2 proteins binding to probes specifically. When a mutant biotin-labeled probe deleted of −289 bp to −275 bp region was added, the shifted band was disappeared (Fig 5E and 5F). However, we observed a band larger than the shift band possibly due to the nonspecific binding of the mutant probe to the cell lysate. Furthermore, a super-shift band that represents the DNA-protein complex was detected when anti-His antibody was added, indicating that there is one binding site on the *Per1* promoter for Rel1 and Rel2.

### The IMD pathway plays a major role in regulating *Per1* expression

**Fig 6:**
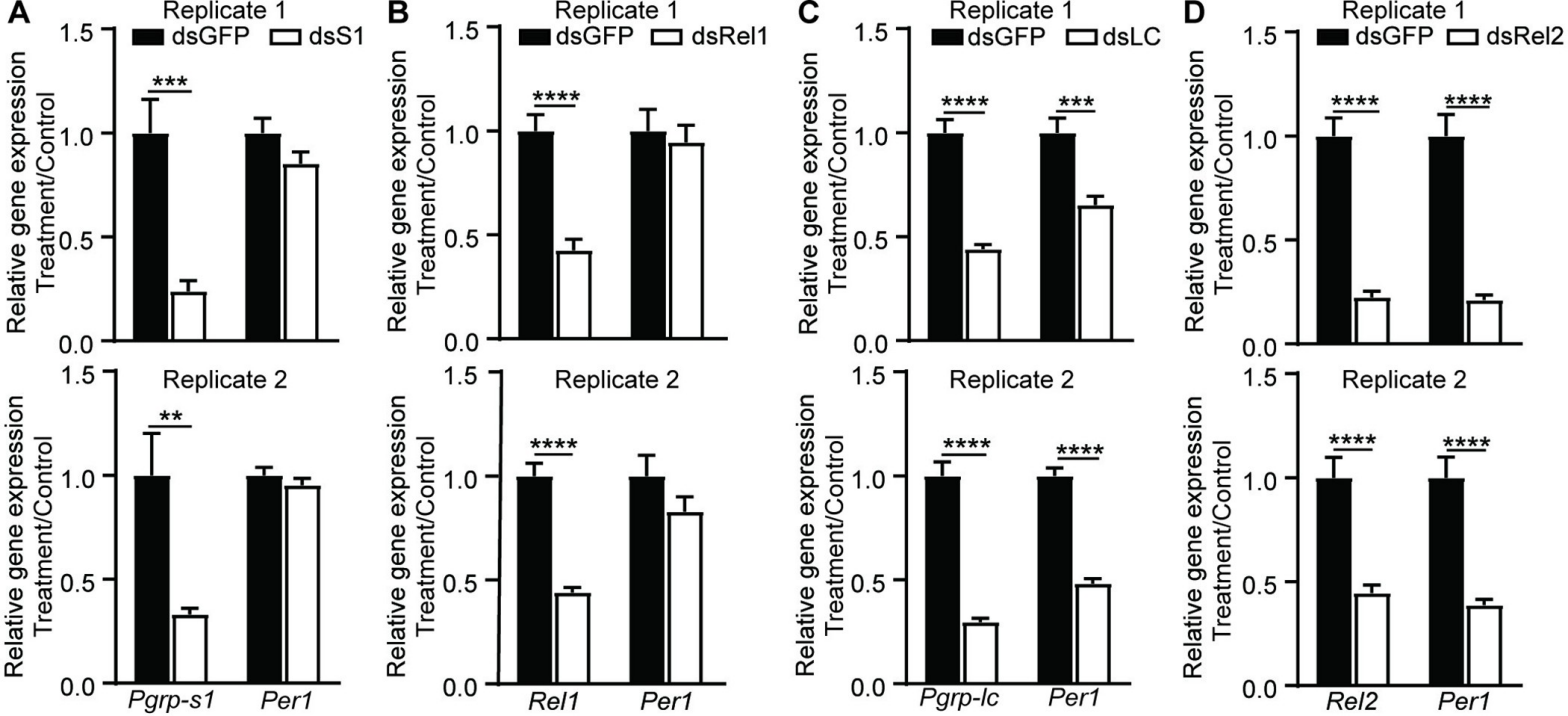
The impact of single gene knockdown in *Per1* expression. The gene knockdown efficiency and *Per1* expression levels in *Pgrp-s1* (A), *Rel1* (B), *Pgrp-lc* (C), and *Rel2* (D) knockdown mosquitoes. Data are presented as mean ± SEM (*n* = 9~10 in A, *n* = 10 in B, *n* = 10 in C, *n* = 8~10 in D). Significance was determined by Student’s *t* test. **, *P* < 0.01, ***, *P* < 0.001, ****, *P* < 0.0001. The data underlying this figure can be found in S1 Data.

As Toll and IMD pathways play different roles in maintaining gut microbial homeostasis and defending against pathogens [47], we next investigated the individual contributions to PM formation. We knocked down *Pgrp-s1*, *Pgrp-lc*, *Rel1*, and *Rel2* individually and analyzed *Per1* expression levels (Fig 6). Knockdown of *Pgrp-s1* or *Rel1*, associated with the Toll pathway, had no effect on *Per1* expression (Fig 6A and 6B). In contrast, specific knockdown of *Pgrp-lc* and *Rel2*, key components of the IMD pathway, significantly down-regulated *Per1* expression (Fig 6C and 6D). Consistent with the stronger effect of DAP-PGN on *Per1* expression (Fig 2C), these findings strongly suggest that IMD pathway plays a major role in regulating PM formation.

## Discussion

The PM, similar to mammalian gut mucus, acts as a physical barrier that protects the insect midgut epithelium from damage. Symbiotic bacteria in hematophagous vectors, such as mosquitoes, ticks, and tsetse flies, play important roles in maintaining PM integrity; however, the underlying mechanisms remain unclear [19,22,23]. Our study revealed that PGNs and LPS are key regulators of PM formation. In the mosquito midgut, the recognition of LPS and PGNs by PGRPs triggers the activation of innate immune signaling pathways. Among these pathways, the IMD pathway plays a predominant role in stimulating PM formation by initiating *Per1* expression, which is essential for PM development.

The bacterial cell wall contains typical pathogen-associated molecular patterns (PAMPs) that activate host immune responses through pathogen recognition receptors (PRRs) [44,48]. For example, DAP-PGN, mostly derived from gram-negative bacteria triggers the IMD pathway that results in Relish activation and robust gene expression of antimicrobial peptide in *Drosophila* [27,49]. While Lys-PGN, derived from gram-positive bacteria, has a higher affinity to PGRP-SA and activates the Toll pathway [50]. In addition, LPS on the surface of gram-negative bacteria cell wall also activates the *Drosophila* immune system, albeit with weaker effects [50]. Beyond immune activation, PGN and LPS also play critical roles in regulating insect physiology. For example, DAP-PGN modulates *Drosophila* female egg-laying behavior by activating the NF-κB pathway in octopaminergic neurons [51–53]. Systemic infection with PGN derived-from *Pseudomonas aeruginosa* reduces the sperm viability of *Drosophila melanogaster* [54]. LPS suppresses feeding and egg-laying behaviors in *Drosophila* to prevent infection by stimulating gustatory neurons via a TRPA1-dependent cation channel [55,56]. Furthermore, LPS induces grooming behavior by activating wing chemoreceptors, helping flies defend against pathogens and parasites [57]. In this study, we demonstrate a novel role of PGN and LPS in stimulating PM formation, thereby enhancing physical barrier function in mosquitoes. These findings suggest that, in addition to acting as PAMPs, LPS and PGN serve as signaling molecules regulating various physiological processes in insects. However, this study focused exclusively on typical cell wall components, PGNs and LPS, in PM formation. Investigating the roles of other bacterial cell wall components, such as teichoic acid, lipoteichoic acid, or unusual structural components, in PM formation will be an important area for future research.

The Toll and IMD pathways are key immune signaling pathways that protect insects from pathogens infection and regulate gut microbiota homeostasis through NF-κB transcription factors [47,58]. In mosquitoes, Rel1, a homolog of *Drosophila* Dorsal, function as a Toll pathway transcription factor, regulating the synthesis of immune effectors [59]. Rel2, the mosquito equivalent of *Drosophila* Relish governs the IMD pathway [60–62]. Unlike *Drosophila* IMD pathway that primarily responds to gram-negative bacteria [63], the mosquito IMD pathway responds to both gram-positive and gram-negative bacteria [47] and serves as the predominant immune pathway in the midgut [64]. This pathway also plays a crucial role in *Plasmodium* defense in mosquitoes. Suppressing IMD activity significantly increases mosquito susceptibility to *Plasmodium* infection, while its activation reduces susceptibility in *Anopheles* mosquitoes [43,61,65].

Beyond their canonical roles in immune regulation, the IMD and Toll pathways also regulate other essential physiological processes in *Drosophila*. For instance, the Toll pathway contributes to epithelial wound repair by controlling E-cadherin expression, a critical component of adherens junctions [66]. It also plays a key role in regulating aging and lifespan in *Drosophila* neural cells [67]. Similarly, the IMD pathway is involved in salivary gland degradation during *Drosophila* larval development by regulating autophagy [68]. In this study, we demonstrate that the IMD pathway plays a predominant role in contributing to PM formation by regulating *Per1* expression, which is consistent with its major role in immune response in the midgut. Additionally, *Per1* has been reported to be regulated by the JAK/STAT signaling pathway in *Ixodes* ticks [19]. Given the diversity of gut microbiota, it is plausible that multiple signaling pathways collectively regulate the expression of *peritrophin* genes, thereby maintaining PM integrity.

In this study, we focused exclusively on the influence of bacteria on PM formation. Given that the PM plays diverse roles in mosquito physiology, such as facilitating blood digestion, defending against pathogens, and maintaining homeostasis [5,69], further research is needed to explore how bacteria-mediated PM formation impacts mosquito biology. In summary, our findings uncover the molecular basis of a noncanonical regulatory role for bacterial cell wall components, LPS and PGNs, in promoting PM formation. Additionally, we highlight an atypical role for immune signaling pathways in maintaining gut barrier integrity. These insights provide a foundation for understanding the intricate interactions between gut microbiota and mosquito physiology.

## Materials and methods

### Mosquito rearing and antibiotic treatment

*An*. *stephensi* (Strain Hor) larvae were reared in water that was supplemented with fish food daily. Adult mosquitoes were kept in a controlled environment at 28°C, 80% relative humidity and at a 12 h light/dark cycle according to standard procedures [70,71]. They were provided with a 10% sugar solution and BALB/c mice for blood feeding. For antibiotic treatment, newly emerged mosquitoes were given fresh filtered 10% sucrose containing 10 U/ml penicillin, 10 μg/ml streptomycin, and 15 μg/ml gentamicin daily for up to 5 days. To determine the efficacy of antibiotic treatment, mosquitoes were surface sterilized with 75% ethanol twice and 0.9% NaCl twice, then homogenized in 0.9% NaCl. The homogenate was plated on the LB agar plates. CFU were counted after incubating the plates at 28°C for 2 days and 5 days. The genomic DNA was extracted using the method of Holmes and Bonner as described [72] and the bacteria load was determined by qPCR using universal 16S rRNA primers (S1 Table). After removing antibiotics, the mosquitoes were reared in sterilized cages and provided with sterilized sucrose solution to maintain a sterile environment [73].

### Bacterial cell wall purification

The purification procedure of bacterial cell wall was performed as described [74]. Briefly, the *E*. *hormaechei* was grown in LB medium at 28°C overnight. Then, 1 × 10^7^–10^8^/ml bacterial cells were harvested by centrifugation at 5, 000 g for 10 min at 4°C. The pellets were collected, washed with distilled endotoxin-free water, and boiled in 8% sodium dodecyl sulfate (SDS) (Sigma-Aldrich) for 30 min. Then, the cells were incubated at room temperature overnight. Afterward, they were centrifuged at 25,000 g for 20 min at 4°C. The resulting cell pellets were resuspended in 4% SDS and boiled for 15 min. Following this, the cell wall was harvested by centrifugation at 25,000 g for 20 min at 4°C and resuspended in distilled endotoxin-free water. This process was repeated 3 times. Finally, the pellets were resuspended in 2 M NaCl and collected by centrifugation at 25,000 g for 20 min at 4°C. The supernatant was discarded and the remaining pellets were dissolved in 10% sucrose or blood for oral feeding mosquitoes.

### Bacterial contents preparation

Briefly, the *E*. *hormaechei* was grown in LB medium at 28°C overnight. Then, 1 × 10^7^–1 × 10^8^/ml bacterial cells were harvested by centrifugation (5,000 g, 10 min, 4°C). The supernatant was discarded, and the cell pellets were washed 3 times with sterile 1× phosphate-buffered saline (1× PBS). The cell pellets were then resuspended in PBS and sonicated on ice in the presence of protease inhibitor (Beyotime). Cell contents were obtained by centrifugation at 143,000 g for 1 h at 4°C and filtered through a 0.22 μm membrane. Then, the filtrate was used for oral administration to mosquitoes via blood meal [75,76].

### Oral administration cell components of bacteria

The oral administration of *E*. *hormaechei* was performed as described [77]. Briefly, the overnight culture *E*. *hormaechei* was harvested by centrifugation (5,000 g, 10 min, 4°C). The pellet and the supernatant were collected. The pellet was washed 2 times with PBS and dissolved in 1.5% sucrose to get the final concentration 1 × 10^7^–10^8^/ml. The supernatant was filtered through a 0.22 μm membrane before adding to 1.5% sucrose. The commercial Lys-PGN derived from *Staphylococcus aureus* (Sigma-Aldrich), DAP-PGN derived from *Bacillus subtilis* (Sigma-Aldrich), and LPS derived from *E*. *coli* (Sigma-Aldrich) were dissolved in sterile water and were administrated to antibiotics treated mosquitoes through a blood meal or 10% sucrose at dedicated concentration. To eliminate the PGN contamination in LPS, the LPS (1 mg/ml) (Sigma) were incubated with mutanolysin (Sigma) at 22°C for 12 h with shaking [78]. The mutanolysin-treated LPS was then orally administrated to Abx-treated mosquitoes through an artificial membrane-feeding system (Hemotek). The mosquito midgut was dissected at 24 h post blood feeding, and the gene expression of *Per1* was analyzed by qPCR.

### Immunofluorescence analysis

The immunofluorescence analysis of Per1 was performed as previously described [14]. Briefly, mosquito abdomens were collected 45 h after a blood meal and fixed in 4% paraformaldehyde at 4°C overnight. Samples were paraffin-embedded, sectioned at thickness of 5 μm, and stained with anti-Per1 (1:100) and Alexa Fluor 546 (1:5,000) (Thermo Fisher). Images were captured using a Nikon ECLIPSE IVi microscope connected to a Nikon DIGITAL SIGHT DS-U3 digital camera.

### Calcofluor white staining

Briefly, mosquito abdomens were collected 45 h after a blood meal and fixed in 4% paraformaldehyde at 4°C overnight. Samples were embedded in paraffin and sectioned at 5 μm thickness. After deparaffinization, the sections were washed 3 times with PBS and then stained with calcofluor white (Sigma) at room temperature for 30 min in the dark. After staining, the sections were rinsed 3 times with distilled water. The sections were then dried and images were captured using a Nikon ECLIPSE IVi microscope connected to a Nikon DIGITAL SIGHT DS-U3 digital camera.

### RNA isolation, cDNA synthesis, and quantitative PCR (RT-qPCR)

Total RNA was extracted from individual mosquitoes using the standard TRIzol reagent (Sigma-Aldrich). The cDNA was synthesized using the Hifair III 1st Strand cDNA Synthesis SuperMix for qPCR (Yeasen). The expression level of the target genes were determined by qPCR using a Roche LightCycler 96 Real Time PCR Detection System with SYBR Green qPCR Master Mix (Yeasen). Data were processed and analyzed using LightCycler 96 software. The ribosomal gene *S7* of *An*. *stephensi* was used as an internal reference [79]. The relative expression of the target gene was normalized to *S7* by the 2^-△△Ct^ method [80]. Seven to 10 mosquitoes were used for qPCR analysis each time. Primers were listed in supplementary S1 Table.

### Western blot analysis

Mosquito midguts were dissected at 45 h post-blood meal and at least 10 midguts were pooled together. Proteins were extracted using lysis buffer (8 M urea, 2% SDS, 5% β-mercaptoethanol, 125 mM Tris-HCl). Immunoblotting was performed using standard procedures with rabbit anti-Per1 polyclonal antibody (1:5,000) [81], anti-Actin monoclonal antibody (1:2,000) (Abbkine), and secondary anti-rabbit-HRP (1:5,000) (Abbkine). Actin was used as a reference control [14,81]. Intensity of the signals was quantified by ImageJ software [82].

### RNA interference

The cDNA clones of target genes were obtained using gene-specific primers (S1 Table). PCR amplicons of GFP (as a control) (BD Biosciences), *Pgrp-lc* (ASTE016447), *Pgrp-s1* (ASTE007708), *Rel1* (ASTE011378), *Rel2* (ASTE010360) tailed with a T7 promoter (TAA TAC GAC TCA CTA TAG GGA GA) were used to synthesize dsRNA using a MEGAscript T7 High Yield Transcription Kit (Invitrogen). Four- to 5-day-old females were received a total of 69 nL dsRNA (3 to 4 μg/μl), which was injected intra-thoracically using a nanoject II microinjector (Drummond). Gene silencing efficiency was determined 2 days after injection. All experiment was replicated twice with separate cohorts of mosquitoes.

### Plasmid construction

The promoter sequence, located 1,589 bp upstream of the *Per1* coding sequence, and the different truncations of the *An*. *stephensi Per1* promoter were amplified using the primers listed in S1 Table. The resulting PCR products were then cloned into plasmid pGL3-Basic vectors carrying the firefly luciferase reporter (Promega). The RHD of *Rel1* and *Rel2* were amplified from an *An*. *stephensi* cDNA using the primers listed in S1 Table, and then cloned into pCMV6-AC-HA vectors (OriGene), named as pCMV6-Rel1-RHD, and pCMV6-Rel2-RHD, respectively. The pRL-TK plasmid carrying renilla luciferase were used as an internal control (Promega). The 4 mutations of the *Per1* promoter (M1-M4) were constructed by amplifying the 489 bp promoter region (pGL3-489) using the primers listed in S1 Table. Plasmids for transfection were prepared using the EndoFree Maxi Kit Plasmid Kit (TIANGEN).

### Dual luciferase assay

Approximately 0.5 million 293T cells were seeded per well in 24-well plates. Once the cells reached almost 70% confluence, the pGL3-Per1, PCMV6-Rel1-RHD-His, pCMV6-Rel2-RHD-His plasmids, and pRL-TK plasmids were transfected into the 293T cells using Hieff Trans Liposomal Transfection Reagent (Yeasen). To investigate the influence of Rel2 and Rel1 on *Per1* expression, the pGL3-Per1, PCMV6-Rel1-RHD-His, pCMV6-Rel2-RHD-His, and pRL-TK plasmids were transfected into the 293T cells. At 48 h post transfection, cells were harvested and lysed with 100 μl per well passive lysis buffer (PLB) supplied with the Dual-Luciferase Report System Kit (Promega). The lysates were centrifuged, and the supernatant was transferred into a 96-well white polystyrene assay plate (Corning). The firefly and renilla luciferase signals were detected according to the manufacturer’s instructions for the Dual Luciferase Report Assay System (Promega) using the GloMax 96 Microplate Luminometer (Promoga). The value of firefly luciferase was normalized by that of renilla luciferase.

### Electrophoretic mobility shift assay (EMSA)

The 293T cells were transfected with a 1:1 mix of pCMV6-Rel1-RHD-His and pCMV6-Rel2-RHD-His plasmids. After 48 h, the transfected cells were harvested and lysed using 200 μl of cell lysis buffer (Beyotime) containing protease inhibitor PMSF (phenylmethanesulfonyl fluoride) (Beyotime) with the following components: 20 mM Tris (pH 7.5), 150 mM NaCl, 1% Triton X-100, sodium pyrophosphate, β-glycerophosphate, EDTA, and Na3VO4. The lysates were centrifuged at 1,3000 rpm for 15 min at 4°C. The resulting supernatant was transferred to a clean, pre-chilled tube and stored at −80°C until use. The EMSA assay was performed using a Light Shift chemiluminescent EMSA Kit (Thermo) according to the manufacturer’s instructions. Briefly, the protein extracts, biotin-labeled oligonucleotide probes and anti-His antibody (Abmart), were added to a 20 μl reaction system containing 1× binding buffer (1 mM Tris, 50 mM KCl, 1 mM DTT; pH 7.5), 0.05% NP-40, 2.5% glycerol, and 50 ng/μl poly (dI:dC). Unlabeled or mutant oligonucleotides were used as the competitors. After incubating at room temperature for 20 min, the reaction mixture was run on a 5% polyacrylamide gel, and then transferred to a Nylon membrane (Bio-Rad) using a Transblot R SD Simi-Dry Transfer System (Bio-Rad). The DNA was then cross-linked to the membrane for 15 min. The membrane was incubated in 20 ml of blocking buffer for 15 min. Subsequently, it was replaced it with another 20 ml of blocking buffer containing 66.7 μl of stabilized streptavidin HRP conjugate (1:300 dilution) for 15 min. After washing 4 times, the membrane was transferred to a new container with 30 ml of substrate equilibration buffer and incubated for 5 min. After being treated with a substrate working solution provided by the EMSA kit, the membrane was imaged using a ChemiDocTM Imaging System (Bio-Rad).

### Statistical analysis

All statistical analyses were performed using GraphPad Prism software (v.8). Details of statistical methods were described in the figure legends. Differences in gene expression between 2 groups were analyzed using the Student’s *t* test, or using analysis of one-way ANOVA followed by Dunnett’s multiple comparison test for more than 2 groups. The microbiota levels of mosquitoes between 2 groups were analyzed using the Mann–Whitney test or using analysis of one-way ANOVA followed by Dunnett’s multiple comparison test for more than 2 groups.

## Supporting information

S1 Fig Bacterial clearance efficacy in *An*. *stephensi*. **(A)** Quantification of midgut bacterial loads by qPCR in the normal (Normal) and antibiotic-treated (Abx) mosquitoes 5 days after antibiotic treatment. (B) CFU of midgut bacteria in normal (Normal) and antibiotic-treated (Abx) mosquitoes growing on LB plates for 5 days. Data are presented as mean ± SEM (*n* = 10 in A, *n* = 10 in B). Significance was determined by Mann–Whitney test. ****, *P* < 0.0001. The data underlying this figure can be found in S1 Data.
(TIF)

S2 Fig The influence of LPS on *Per1* expression. The LPS was treated with mutanolysin and orally supplemented to mosquitoes. The mutanolysin-untreated LPS and mutanolysin-supplemented mosquitoes were used as controls. Data are presented as mean ± SEM (*n* = 10). Significance was determined by one-way ANOVA followed by Dunnett’s multiple comparison test. **, *P* < 0.01. The data underlying this figure can be found in S1 Data.
(TIF)

S3 Fig The influence of DAP-PGN/Lys-PGN/LPS mixture on other PM genes expression. (A, B) The expression level of *Per14* (A) and *Fibrinogen* (B) in the midgut of Normal, Abx, Abx mosquitoes treated with DAP-PGN/Lys-PGN/LPS (PGNs+LPS) for 24 h via sugar meal. Data are presented as mean ± SEM (*n* = 10 in A, *n* = 10 in B). Significance was determined by one-way ANOVA followed by Dunnett’s multiple comparison test. *, *P* < 0.05, ***, *P* < 0.001. The data underlying this figure can be found in S1 Data.
(TIF)

S4 Fig PM gene and microbiota regulation by Rel1 and Rel2. The expression levels of *Per14* (A) and *Fibrinogen* (B) in dsRel1/Rel2 and dsGFP-treated mosquitoes prior to blood feeding. (C) The total gut microbiota load was measured in dsRel1/Rel2 and dsGFP-treated mosquitoes post blood feeding. Data are presented as mean ± SEM (*n* = 8~9 in A and B, *n* = 8~10 in C). Significance was determined by Student’s *t* test in A and B and by Mann–Whitney test in C. *, *P* < 0.05, ***, *P* < 0.001. The data underlying this figure can be found in S1 Data.
(TIF)

S1 Raw Images PDF file with all the original, uncropped blot and gel images corresponding to the data presented in the article. (TIF)

S1 Data Raw data supporting Figs 1–6 and S1–S4. (XLSX)

S1 Table List of primers used in the article. (DOCX)

## Abbreviations

AMP: antimicrobial peptide
CBD: chitin-binding domain
CFU: colony-forming unit
DAP-PGN: diaminopimelic acid-peptidoglycan
EMSA: electrophoretic mobility shift assay
LPS: lipopolysaccharide
Lys-PGN: lysine-peptidoglycan
PAMP: pathogen-associated molecular pattern
PBS: phosphate-buffered saline
PLB: passive lysis buffer
PM: peritrophic matrix
PMSF: phenylmethanesulfonyl fluoride
PRR: pathogen recognition receptor
RHD: Rel-homology domain
SDS: sodium dodecyl sulfate
