# Supplementary figures and images for: Cell wall components of gut commensal bacteria stimulate peritrophic matrix formation in malaria vector mosquitoes through activation of the IMD pathway
Source: PLoS Biol. 2025 Jan 6;23(1):e3002967. doi: 10.1371/journal.pbio.3002967 (PMC11703001; doi:10.1371/journal.pbio.3002967)

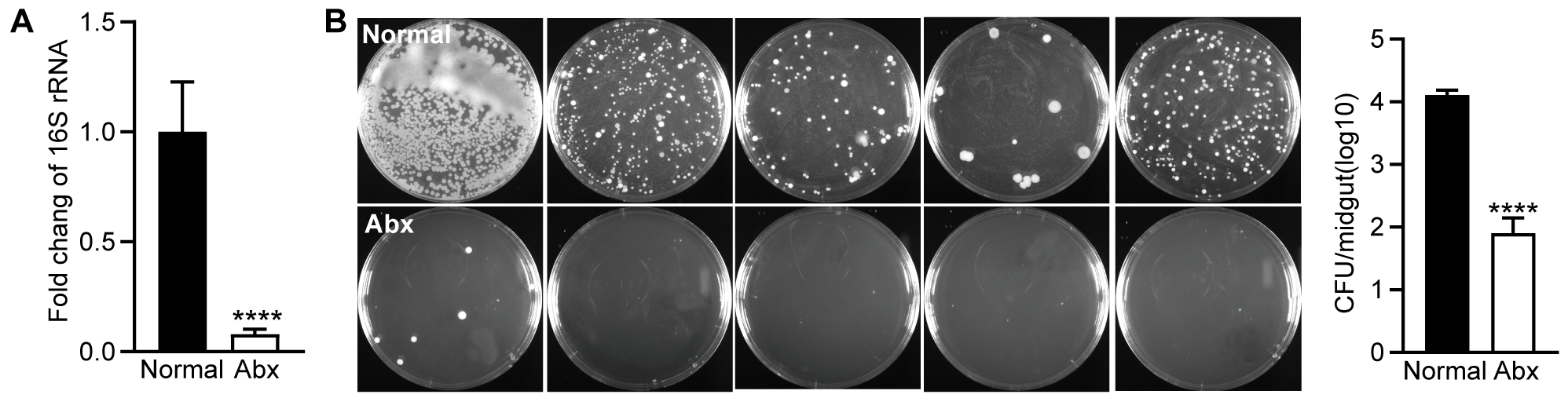

Supplement: S1 Fig — (A) Quantification of midgut bacterial loads by qPCR in the normal (Normal) and antibiotic-treated (Abx) mosquitoes 5 days after antibiotic treatment. (B) CFU of midgut bacteria in normal (Normal) and antibiotic-treated (Abx) mosquitoes growing on LB plates for 5 days. Data are presented as mean ± SEM (n = 10 in A, n = 10 in B). Significance was determined by Mann–Whitney test. ****, P < 0.0001. The data underlying this figure can be found in S1 Data. (TIF) [file pbio.3002967.s001.tif]

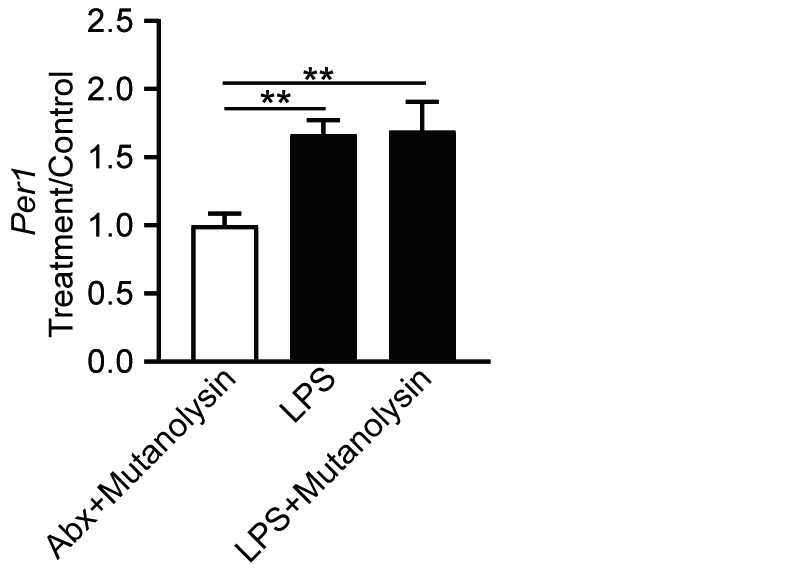

Supplement: S2 Fig — The LPS was treated with mutanolysin and orally supplemented to mosquitoes. The mutanolysin-untreated LPS and mutanolysin-supplemented mosquitoes were used as controls. Data are presented as mean ± SEM (n = 10). Significance was determined by one-way ANOVA followed by Dunnett’s multiple comparison test. **, P < 0.01. The data underlying this figure can be found in S1 Data. (TIF) [file pbio.3002967.s002.tif]

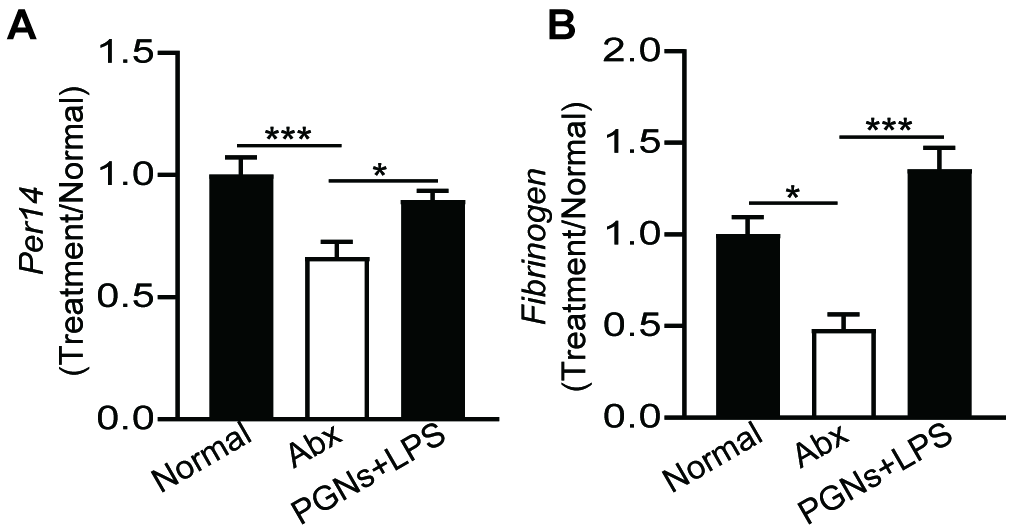

Supplement: S3 Fig — (A, B) The expression level of Per14 (A) and Fibrinogen (B) in the midgut of Normal, Abx, Abx mosquitoes treated with DAP-PGN/Lys-PGN/LPS (PGNs+LPS) for 24 h via sugar meal. Data are presented as mean ± SEM (n = 10 in A, n = 10 in B). Significance was determined by one-way ANOVA followed by Dunnett’s multiple comparison test. *, P < 0.05, ***, P < 0.001. The data underlying this figure can be found in S1 Data. (TIF) [file pbio.3002967.s003.tif]

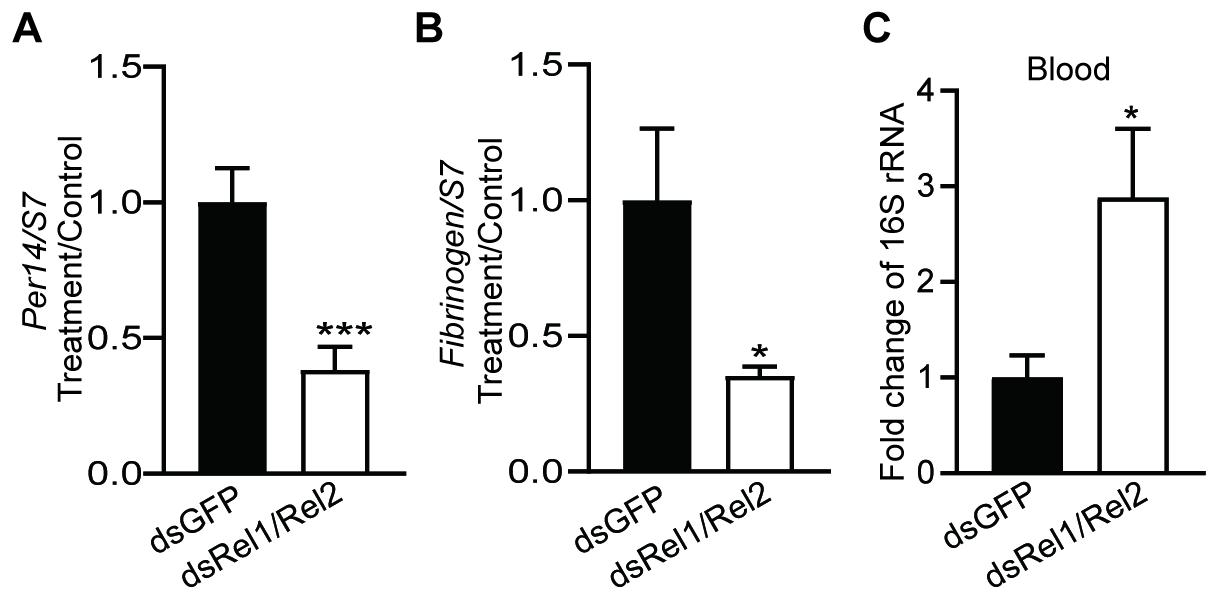

Supplement: S4 Fig — The expression levels of Per14 (A) and Fibrinogen (B) in dsRel1/Rel2 and dsGFP-treated mosquitoes prior to blood feeding. (C) The total gut microbiota load was measured in dsRel1/Rel2 and dsGFP-treated mosquitoes post blood feeding. Data are presented as mean ± SEM (n = 8~9 in A and B, n = 8~10 in C). Significance was determined by Student’s t test in A and B and by Mann–Whitney test in C. *, P < 0.05, ***, P < 0.001. The data underlying this figure can be found in S1 Data. (TIF) [file pbio.3002967.s004.tif]

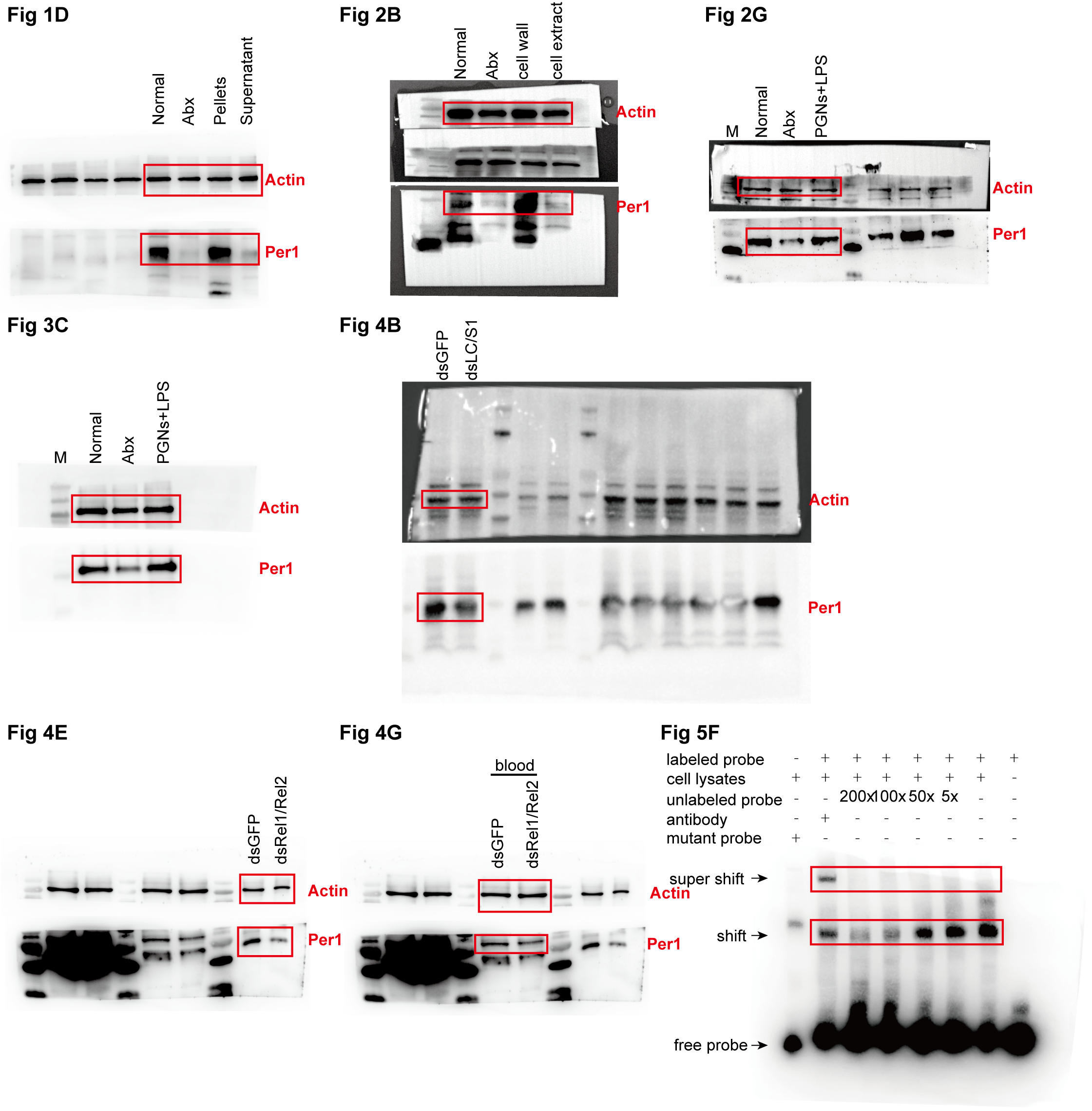

Supplement: S1 Raw Images — (TIF) [file pbio.3002967.s005.tif]
