# Supplementary material for: Cell wall components of gut commensal bacteria stimulate peritrophic matrix formation in malaria vector mosquitoes through activation of the IMD pathway
Source: PLoS Biol. 2025 Jan 6;23(1):e3002967. doi: 10.1371/journal.pbio.3002967 (PMC11703001; doi:10.1371/journal.pbio.3002967)
Supplement: S1 Table — (DOCX) [file pbio.3002967.s007.docx]

| **S1 Table: Primer sequences** | |
| --- | --- |
| Primer | Sequence (5’ to 3’) |
| qS7-F (ASTE004816) | TGCGGAGCGTCGTATTCTGC |
| qS7-R | ACACAGCGGTGAGCGTTCG |
| qPGRP-LC-F (ASTE016447) | TTGCGGCTGGTGACGAGAAC |
| qPGRP-LC-R | CCCTTCGGTGGCAGTGTGAG |
| qPGRP-S1-F (ASTE007708) | CGAGACGGGTGAACTTGCTGA |
| qPGRP-S1-R | GTCCTCCTGCCAGTGGTCAA |
| qPer1-F(ASTE010406) | AGCCACGGCAATGCGGTTGT |
| qPer1-R | TCGGACGGTTGCAGCGGTTG |
| qRel1-F  (ASTE011378) | AGGTTGCCATCAGTTTCCGA |
| qRel1-R | TCCGAGTAGGTGTTGTCCGA |
| qRel2-F  (ASTE010360) | AACAGCAGCAACAGCATTACT |
| qRel2-R | TGATAGCGGAAGCGGAACT |
| qDefensin-F  (ASTE011281) | CCGCCTTGAACACGCTCCT |
| qDefensin-R | GCTGCCGACACCGAATCCA |
| qGambicin-F  (ASTE002252) | CCGCTGTTCGTCCTCGTTCA |
| qGambicin-R | GCACACGGACGCCAACTTCT |
| qCecropin-F  (ASTE007106) | GGTAGATGGTGCGCCCCGTT |
| qCecropin-R | GGTAGATGGTGCGCCCCGTT |
| qPer14-F  (ASTE009456) | TGGTGCTCGCTACGTTCGCT |
| qPer14-R | TGCAAACCGCCGGGACAGT |
| qFibrinogen-F  (ASTE010194) | TTTGGCTGGGGTTGGATCGT |
| qFibrinogen-R | CCTGCCGTGCCCGAATAGC |
| GFP-T7-F  (BD Biosciences) | TAATACGACTCACTATAGG GTCAGTGGAGAGGGTGAAG |
| GFP-T7-R | TAATACGACTCACTATAGG CTAGTTGAACGGATCCATC |
| T7-PGRP-LC-F | TAATACGACTCACTATAGG GCAGCAACGGTGGCAGCGATAC |
| T7-PGRP-LC-R | TAATACGACTCACTATAGG TGACCGCTACGATGGCACACAGAAC |
| T7-PGRP-S1-F | TAATACGACTCACTATAGG GGCAGGATGATGGTTCTA |
| T7-PGRP-S1-R | TAATACGACTCACTATAGG GATAGTCGTCAGCAAGTTC |
| T7-Rel1-F | TAATACGACTCACTATAGG CACAAGCCTCATCCGCACAATCTGG |
| T7-Rel1-R | TAATACGACTCACTATAGG CCTTCACCACCTTCTCGCACAGCAA |
| T7-Rel2-F | TAATACGACTCACTATAGG AACGGCCGGTGACGATAGT |
| T7-Rel2-R | TAATACGACTCACTATAGG TTAGTAATCCGTCCCGTTC |
| 16S rRNA 27-F | agagtttgatcctggctcag |
| 16S rRNA 1492-R | catgctgcctcccgtaggagt |
| Rel2-RHD-F (BamH I) | GAATTCGTCGACTGGATCCGCCACCATGGCCAAACCGCATCTGGTGATACTG |
| Rel2-RHD-R (Not I) | GGGTACTCGAGCGGCCGC TTA ATGATGATGATGATGATGGCGCGGTTTGTACTTAAACAG |
| Rel1-RHD-F (BamH I) | GAATTCGTCGACTGGATCC GCCACCATGGCC CCGTACGTGGAGATCACGGA |
| Rel1-RHD-R (Not I) | GGGTACTCGAGCGGCCGCTTAATGATGATGATGATGATGGTCTAGTGGTATGAACTGGAA |
| Per1-promoter-F | TTAATTGATTAGTGCCGATCAT |
| Per1-promoter-R | GATGAGAATGTTAGATGCCGCG |
| pGL3-1589-F (Nhel) | TACGCGTGCTAGCC TGTGTCGAACTGATAACACCC |
| pGL3-689-F (Nhel) | TACGCGTGCTAGCC ACGGCTTGTGTGCCTATCGGAA |
| pGL3-489-F (Nhel) | TACGCGTGCTAGCC TTCATAAAATCGAATGAAGGAT |
| pGL3-289-F (Nhel) | TACGCGTGCTAGCC ATAGACACGAGCACCGTGCTGA |
| pGL3-229-F (Nhel) | TACGCGTGCTAGCC TGTCCCACCA TCCAAGCAGCG |
| pGL3-Per1-R (Bglll) | CAAGCTTACTTAGATCGCAGATCT TGCAACAGGTACAGTACGTAA |
| pGL3-M1-F | TTAGTAGATCGCCAAGTGCTGAGATTCACT |
| pGL3-M2-F | ATAGACACGAGCACCTTTGATAGCCGATAG |
| pGL3-M3-F | GTGCTGAGATTCACTTCGATAAGTTGAAAG |
| pGL3-M4-F | TTTGATAGCCGATAGTGTCCCACCA |
| pGL3-Per1-R | CAAGCTTACTTAGATCGCAGATCT TGCAACAGGTACAGTACGTAA |
| M1-F (bio) | TTAGTAGATCGCCAAATAGACACGAGCACCGTGCTGAGATTCACT |
| M1-R (bio) | AGTGAATCTCAGCACGGTGCTCGTGTCTATTTGGCGATCTACTAA |
| M1-F | TTAGTAGATCGCCAAATAGACACGAGCACCGTGCTGAGATTCACT |
| M1-R | AGTGAATCTCAGCACGGTGCTCGTGTCTATTTGGCGATCTACTAA |
